# A Case of Appendicitis

**Published:** 1893-07

**Authors:** William Hunt


### A CASE OF APPENDICITIS.

BY WILLIAM HUNT, M. D.

This specimen was removed from a young girl who presented every sign of good health, but had been sent to the Pennsylvania Hospital by a physician outside, who had diagnosed possible stone in the bladder from the general symptoms. She was examined by the residents on admission, and they thought that they felt a stone. I saw the patient but once and I examined her for stone, but did not find one. I decided to let her rest and to repeat the examination under ether the following day. At three o'clock she was taken with severe pain in the lower part of the abdomen. At eight o'clock she seemed to be doing well, but at eleven o'clock there was a return of the symptoms, and she died in two or three hours.

The coroner's physician was sent for and made an examination, and the death was ascribed to "idiopathic peritonitis." I and Dr. Morton were telephoned to this effect and immediately sent back word to make further examination. It was then found that the appendix was large and swollen and had a large perforation in it. The abdomen was full of pus. It was afterward learned that she had been ailing for two weeks, but there were no symptoms referable to the appendix.

DISCUSSION.

DR. THOMAS G. MORTON: I have seen more than one case where it was impossible to make a diagnosis; the most remarkable instance was the following: In March, 1890, Dr. DaCosta and I were summoned to Trenton, N. J., to see a patient with Dr. Phillips, of that city. The patient was about forty-two years of age; he had been apparently in excellent health three days before; he became obstinately constipated and had nausea and had vomited; little or no abdominal pain and no swelling, a normal temperature, no chills, and pulse showed no acceleration. The bowel simply refused to respond to cathartics. Inquiry showed that there had been no former attack of pain in the appendix region. Calomel and fractional doses of podophyllin were given.

The following day there was a subnormal temperature and great prostration, but no evidence of local or general peritonitis, and the diagnosis could not be cleared up. Median exploratory abdominal section showed a foul abscess in the right iliac fossa; an inflamed gangrenous appendix; the ileum was covered wiih lymph, and for several inches showed structural change. Two days subsequently the patient died.

In reviewing this case I can see no way by which the nature of the case could have been determined; it seems hardly possible that perforative appendicitis and intestinal gangrene should not present at least some positive symptoms, yet in this instance, none such were present.

DR. JOSEPH W. HEARN: I had a case at the Jefferson Hospital where the man had walked to the hospital the evening before, with a temperature of 101°. Dr. Keen saw him the next day, and we agreed that it was a case of appendicitis. Operation was done and a quart of pus evacuated. The man died subsequently.

DR. T. S. K. MORTON: In this connection, as illustrating how patients with very serious disease are able to perform almost incredible exertions, I may mention a case of strangulated hernia that walked to the Polyclinic Hospital to-day—quite a long distance—after his physician had made strong taxis for half an hour. The strangulation had existed five days, and when I operated, the gut was found gangrenous.

---

AMERICAN VS. FOREIGN PHARMACEUTICAL ARTICLES.—Inconsistency is probably the most common failing of mankind. We find a lot of doctors who are unwilling to use an American proprietary article, such as advertised in the medical journals, yet dectors and medical journals take up foreign articles, even when the formula is *secret* and *patented*, and use them.

Why can't an American pharmacist make as good a thing as a German, for instance? Further, the former does'nt talk about a trademark and *conceal his formula.*

This country is getting old enough to let go the leading strings and lead itself. If you can get a good non-secret preparation in America what on earth is the use of importing some patented article from abroad? Be consistent.—*Atlanta Med. and Sur. Journal, May*, 1893.

